# Nodal Merkel Cell Carcinoma with Unknown Primary Site and No Distant Metastasis: A Single-Center Series

**DOI:** 10.3390/cancers14194777

**Published:** 2022-09-29

**Authors:** Nicola Fazio, Patrick Maisonneuve, Francesca Spada, Lorenzo Gervaso, Chiara Alessandra Cella, Marta Pozzari, Dario Zerini, Eleonora Pisa, Caterina Fumagalli, Massimo Barberis, Alice Laffi, Chiara Maria Grana C., Gianmarco Orsolini, Pierpaolo Prestianni, Guido Bonomo, Luigi Funicelli, Emilio Bertani, Paola Queirolo, Davide Ravizza, Manila Rubino, Giulio Tosti, Elisabetta Pennacchioli

**Affiliations:** 1 Division of Gastrointestinal Medical Oncology and Neuroendocrine Tumors, European Institute of Oncology (IEO), IRCCS, 20141 Milan, Italy; 2 Division of Epidemiology and Biostatistics, IEO European Institute of Oncology IRCCS, 20141 Milan, Italy; 3 Molecular Medicine Program, University of Pavia, 27100 Pavia, Italy; 4 Division of Radiotherapy, IEO European Institute of Oncology IRCCS, 20141 Milan, Italy; 5 Department of Pathology and Laboratory Medicine, IEO European Institute of Oncology IRCCS, 20141 Milan, Italy; 6 Humanitas Research Hospital, Rozzano, 20089 Milan, Italy; 7 Division of Nuclear Medicine, IEO European Institute of Oncology IRCCS, 20141 Milan, Italy; 8 Division of Melanoma and Sarcoma Surgery, European Institute of Oncology (IEO), IRCCS, 20141 Milan, Italy; 9 Division of Interventional Radiology, European Institute of Oncology IRCCS (IEO), 20141 Milan, Italy; 10 Division of Medical Imaging and Radiation Sciences, IEO, European Institute of Oncology IRCCS, 20141 Milan, Italy; 11 Division of Digestive Surgery, IEO European Institute of Oncology IRCCS, 20141 Milan, Italy; 12 Division of Medical Oncology of Melanoma, Sarcoma and Rare Tumors, IEO European Institute of Oncology IRCCS, 20141 Milan, Italy; 13 Division of Endoscopy, IEO European Institute of Oncology IRCCS, 20141 Milan, Italy

**Keywords:** Merkel cell carcinoma, unknown primary, Merkel cell polyoma virus, MCC, CUP, MCPyV

## Abstract

**Simple Summary:**

Merkel cell carcinoma is a very rare and highly aggressive neuroendocrine carcinoma originating from the skin. Exceptionally it presents with a nodal localization without a cutaneous primary site and distant metastases. This entity is controversial in terms of origin and clinical management. The main histological differential diagnosis is that of small cell neuroendocrine carcinoma. As a referral center for neuroendocrine neoplasms with more than 20 years of experience we have dealt with patients showing this clinical context several times and we usually manage them within our dedicated multidisciplinary team. Due to the extreme rarity of the entity and undefined clinical management, we report our single-center series and detail some of the diagnostic and therapeutic aspects. Our analysis can be helpful for centers which manage these patients and future investigations on the topic.

**Abstract:**

Merkel cell carcinoma (MCC) is a very rare and aggressive neuroendocrine carcinoma originating from Merkel cells, typically with a skin nodule; however, it exceptionally presents with only a basin lymph node localization, with neither a cutaneous primary site nor distant metastases. From 1996 to 2020, among patients with histologically confirmed MCC managed at a neuroendocrine neoplasm-referral center, we selected those with an exclusive nodal basin, no distant metastasis, and an unknown primary site defined by cross-sectional and physical examination. A total of 55 out of 310 patients fulfilled the selection criteria. The median age was 64 years and the majority were males. Inguinal lymph-nodes were the most common anatomic site. With a median follow-up of 4.3 years, the 5-year relapse-free survival (RFS) rate was 56.6 (95% CI 42.0–68.8%) and the 5-year cancer specific survival (CSS) rate was 68.5 (95% CI 52.8–79.9%) for the whole population. The 36 patients (65.5%) undergoing lymphadenectomy (LND) + radiotherapy (RT) ± chemotherapy had a 5-year RFS rate of 87.2% (95% CI 65.5–95.7%) and a 5-year CSS rate of 90.5% (95% CI 67.0–97.5), which were better than those receiving LND alone. In a multivariable analysis, the survival benefit for LND + RT remained significant. Results from one of the largest single-center series of nMCC-UP suggest that a curative approach including RT can be effective, similar to what is observed for stage IIIB MCC. Multicentric studies with homogenous populations should be carried out in this controversial clinical entity, to minimize the risk of biases and provide robust data.

## 1. Introduction

Merkel cell carcinoma (MCC) was described for the first time as a trabecular carcinoma of the skin in 1972 by Cyril Toker [1]. The term MCC appeared in 1978, with the second description of a trabecular carcinoma [2], and was related to the Merkel cells of the epidermis, described in 1875 by the German pathologist, Friedrick Merkel [3]. However, the origin of MCC cells is currently debated, including dermal fibroblasts, pre/pro B cells and even a pluripotent dermal stem cell [4,5]. Moreover, MCC is considered a neuroendocrine carcinoma (NEC) for several reasons, including the cells’ resemblance to those of the diffuse neuroendocrine system, the immunohistochemical (IHC) expression of chromogranin-A (CgA), synaptophysin (SYN), CD56 and neuron-specific enolase (NSE), and the functional expression of somatostatin receptors (SSTR) during SSTR-imaging (SRI) [6,7]. Additionally, in 2008, a new virus from the polyoma family was discovered, which was specifically linked to MCC and the Merkel cell polyoma virus, (MCPyV), which is detected in most cases. This virus has also been reported to be involved in tumorigenesis [8]. Based on these factors, two different types of MCC have been identified, which are virus-positive (VP) (around 80% of cases [9]) and virus-negative (VN), with clear biological, clinical, and different prognostic features [10,11,12].

Merkel cell carcinoma is an extremely rare disease, accounting for < 1 new case/100,000 per year in Europe and the US, and reaching up to 1.6 cases/100,000 per year in Queensland, Australia. Skin is the usual primary site; however, in rare cases, distant metastatic MCC has been reported with no evidence of a cutaneous primary [13]. Even more rarely, MCC can present with a superficial nodal localization with no evidence of a skin primary site and no distant metastasis, defined as MCC of unknown primary (MCC-UP). To date, this presentation was reported only within case reports or case series accounting for around 200 total cases, with better reported survival outcomes compared to known primary (KP) MCC [14,15,16,17,18,19,20,21,22,23].

In accordance with the American Joint Committee on Cancer (AJCC) TNM 8th edition [24], clinically evident nodal MCC with “no evidence of primary tumor” (T0) and “no distant metastasis” (M0) can be defined as a clinical stage III (anyT cN1-3 M0) or pathological stage IIIA (pT0 N1b or higher M0). Current standard management options for MCC with macroscopically positive lymphnodes include, in addition to radical surgical excision of the primary site and, nodal dissection also RT with 40–50 Gy [9]. The non-metastatic nodal MCC-UP treatment algorithm relies on the same factors, even though it is derived from low quality results.

Due to its heterogeneity, MCC patients may be managed by different specialists, including skin cancer-dedicated or neuroendocrine neoplasm (NEN)-dedicated pathologists, dermatologists, skin cancer surgeons, skin cancer medical oncologists, and NEN-expert medical oncologists. This fragmentation, together with its rarity, is a key limiting factor for the development of concrete evidence for this specific entity, thus leading to undefined clinical management.

On these bases, we assessed a large series of nodal MCC-UP patients with the aim to evaluate therapeutic strategies and clinical/biological factors and to study their relations to survival outcomes.

## 2. Materials and Methods

### 2.1. Patients

Among the 310 patients with MCC examined at the European Institute of Oncology (IEO) between 1996 and 2020, we selected consecutive cases with exclusive single nodal basin involvement, no evidence of cutaneous primary tumor, and no distant metastasis. All patients had received a histological diagnosis of MCC from a nodal basin, performed or confirmed by IEO NEN-referral pathologists. At least one year of follow-up was required. All patients without a clinical evaluation at the IEO in the last six months were contacted to assess patient/tumor status. Treatment could have been performed at the IEO or at another hospital, but the indication (or confirmation) was discussed at the IEO, mostly by a dedicated MDT. This retrospective analysis complies with the Declaration of Helsinki and was approved by our local institutional review board (IRB). A waiver of specific consent was granted due to the study’s retrospective nature.

Several parameters were collected, including age at diagnosis, site of nodal localization, Computed Tomography (CT)/magnetic resonance imaging (MRI), ^18^F-fluorodeoxyglucose (FDG) positron emission tomography (PET)/CT, somatostatin receptor imaging (SRI), date of first visit at the IEO, and treatment.

### 2.2. Pathological Analyses

Two NEN-referral pathologists (E.P., M.B.) independently reviewed all cases to confirm the diagnosis of MCC. All tumor specimens were formalin fixed paraffin embedded (FFPE) and stained with hematoxylin and eosin. The main histologic parameters were collected, including IHC expression of neuroendocrine markers (Chromogranin A, CgA, Synaptofisin, SYN, Neurofilament, NF), epithelial markers (Cytokeratine-20, CK20), virus-positivity marker (Merkel cell polyoma virus, MCPyV large T antigen-LTAg), and others such as the thyroid transcription factor (TTF) 1, and the Ki-67% label index (Clone MIB-1). Expression of MCPyV LTAg in the tumor tissue was assessed using IHC in FFPE MCC samples with a monoclonal antibody (CM2B4, sc-136172; Santa Cruz Biotechnology, Santa Cruz, CA, USA), as recommended by the manufacturer. Tumor cells were considered positive for LTAg when clear nuclear staining, compared to an established positive external control, was seen and present in at least 20% of the entire section.

### 2.3. Statistical Analyses

Differences in the distribution of patients, tumors, or treatments characteristics according to the period of diagnosis were assessed using Fisher’s exact test. Relapse free survival (RFS) was defined as the interval from the date of diagnosis of MCC to the date of recurrence or last contact with the patient. Cancer specific survival (CSS) was defined as the interval from the date of diagnosis to the date of MCC-related death, or last contact with the patient. RFS and CSS curves were drawn using the Kaplan-Meier method and the difference in survival between groups was assessed using the log-rank test. Five-year survival rates with 95% confidence intervals (CI) were calculated using the actuarial method. Univariable and multivariable Cox proportional hazards regression models were used to assess the association between the various characteristics and the risk of recurrence or death from disease. All analyses were carried out with the SAS software version 9.4 (Cary, NC, USA). All statistical tests were two-sided.

## 3. Results

Among the 310 patients who had received a histological diagnosis of MCC, 55 (17.7%) were identified based on the selection criteria. Median age at diagnosis was 64 years, males represented the majority (65.5%)of patients, and the inguinal nodal basin was most frequently involved (78.2%). The median interval between the first clinical appearance of the adenopathy and the first histological diagnosis of MCC was 2.3 months, varying from less than 1 month to more than 1 year (15.4 months) (Table 1). All patients had a baseline CT-scan or MRI.

### 3.1. Tumor Tissue Biomarkers

The median Ki-67 value was 80%, with only three cases with Ki67 < 55%. A total of 49 out of 55 (89.0%) tumors were CK-20 positive, while no cases were positive for TTF-1 (0/45, 10 missing). LTAg was positive in 45/51 (88.2%) and NF was positive in 36/42 (85.7%) (Supplementary Table S1).

### 3.2. Treatment

Thirty-six patients (65.5%) underwent lymphadenectomy (LND). The median number of lymph nodes removed was 18 (range 5–50) with a positive lymph node ratio of 0.21. In 11 patients (30.6%), no malignant lymph node was detected with LND. Twenty-four patients (43.6%) received “adjuvant” radiotherapy (RT) and ten patients (18.2%) received “adjuvant” chemotherapy (ChT), mostly cisplatin/carboplatin + etoposide. Seven patients (12.7%) received both “adjuvant” RT and ChT (Table 2). A higher proportion of patients (57.6% vs. 22.7%) received an LND and “adjuvant” RT in the 2010–2019 group, compared to older patients.

Conversely, less patients received adjuvant ChT in the recent subgroup. The number of lymph nodes removed, and the positive lymph node ratio were similar during the two time periods.

### 3.3. Efficacy

With a median follow-up of 4.3 years (range 2 months to 24 years), 24 patients (43.6%) experienced tumor progression and 20 (36.4%) died (15 due to MCC and 5 due to other causes, including a pancreatic adenocarcinoma and a hematological malignancy).

**Figure 1:**
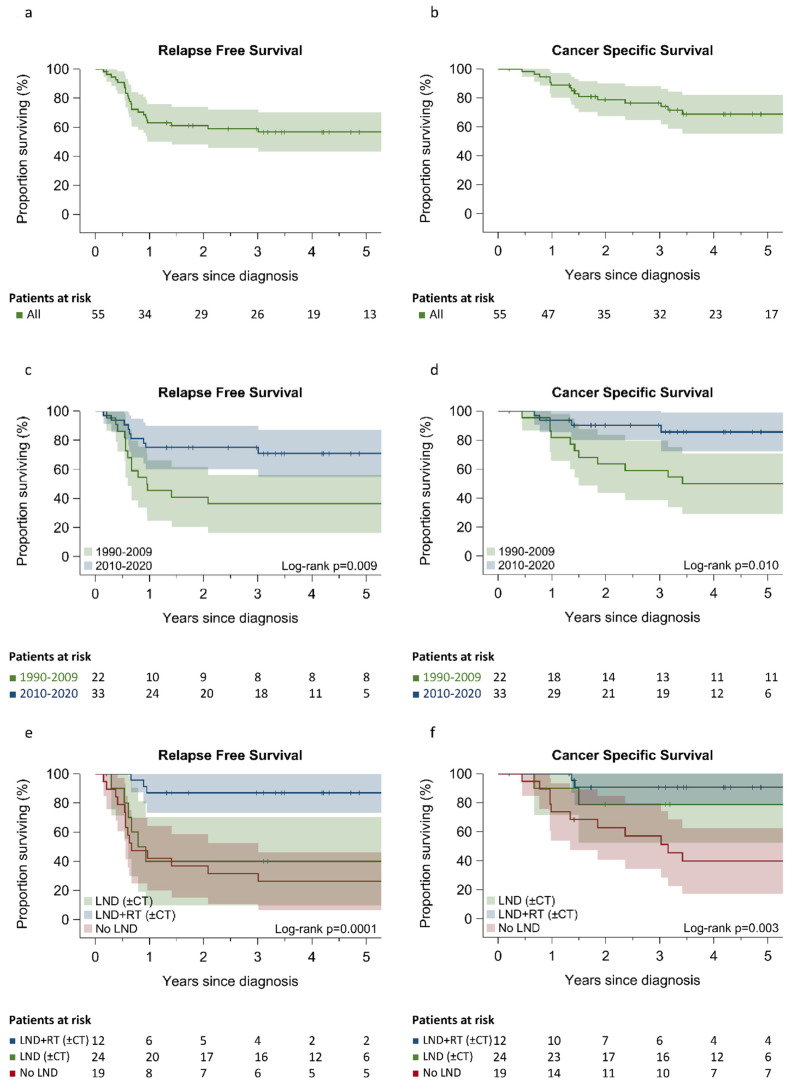
Relapse free survival and cancer –specific survival for all patients (**a**,**b**), by time period (**c**,**d**) and according to treatment modality (**e**,**f**). LND: lymph node dissection; RT: radiotherapy; ChT: chemotherapy.

The 5-year RFS rate was 56.6% (95% CI 42.0–68.8%) and the 5-year CSS rate was 68.5% (95% CI 52.8–79.9%) for the whole patient population. The median RFS and the median CSS were not reached at the end of follow-up (Figure 1a,b). The 5-year RFS and CSS were respectively 70.3% [95% CI 50.0–83.7%] and 84.4% [95% CI 62.4–94.1%] for patients treated later than 2010 vs. 36.4% [95% CI 17.4–55.7%] and 50.0% [95% CI 28.2–68.4%] for patients treated earlier than 2010 (Figure 1c,d).

Among the 36 patients who received potentially curative treatments, those receiving LND + RT (±ChT) had a better 5-year RFS (87.2% [95% CI 65.5–95.7%] and better 5-year CSS (90.5% [95% CI 67.0–97.5%]) than those receiving LND (±ChT) without RT with a 5-year RFS and CSS of 40.0% [95% CI 12.3–67.0%] and 77.5% [95% CI 35.7–93.9%], respectively. The remaining 19 patients who received only palliative treatment had a poor 5-year RFS rate [26.3% (95% CI 9.6–46.8%)] and 5-year CSS rate [39.9% (95% CI 18.2–61.0%)] (Figure 1e,f).

### 3.4. Univariate and Multivariable Analyses

In the univariate analysis, patients diagnosed earlier than 2010 had an approximately 3-fold higher risk of developing a relapse and a 4-fold higher risk of dying due to MCC compared with patients diagnosed later than 2010 (Table 3). Other factors associated with relapse or death included LND, RT and ChT.

In the multivariable analysis, the associations with LND + RT remained. Compared to patients who received LND + RT, patients treated with LND alone had a 6.34-fold higher risk of tumor progression, while patients who received only palliative treatment had a 7.80-fold higher risk of tumor progression and 5.15-fold higher risk of dying of MCC (Table 4).

## 4. Discussion

The results from our large single-center series of nMCC-UP describe a (Supplementary Table S2) 68.5% 5-year CSS rate, which is encouraging compared to the 26% 5-year relative survival rate of KP-MCC with clinically positive regional nodes (cN1 MCC-KP) [25]. This result is in line with previously reported findings, indicating that pathological nodal MCC-UP has a better prognosis than nodal MCC-KP [14,23]. Specifically looking at the cN1 stage, the reported 5-year OS rate was 26.8% for KP-MCC and 42.2% UP-MCC. This information was based on 9387 patients with MCC, according to the AJCC TNM staging system’s 7th [26] and 8th editions. The reason why MCC pT0N1bM0 (Stage IIIA TNM 8th) patients have a better prognosis than those with MCC pT1-4 N1b M0 (Stage IIIB TNM 8th) is still unclear. Moreover, it is also difficult to explain the similar prognosis reported for pT0N1bM0 and pT1-3N1aM0 (both Stage IIIA TNM 8th); it is possibly related to an improved cell-mediated immunity, which clears the primary tumor and controls residual disease. [27].

Merkel cell carcinoma-UP appears not to be related to immune suppression, showing elevated MCPyV oncoprotein antibody titers and a higher number of mutations in the tumor, compared to MCC-KP. This enhanced immune function could facilitate an nMCC-UP by eliminating the cutaneous originating site [28]. A recent report revealed that VP and VN nMCC-UP exhibit an immune-profile similar to that of VP and VN MCC-KP, and that VN nMCC-UP presents UV signatures and a high tumor mutational burden, supporting the probable cutaneous origin of VN nMCC-UP [29]. However, the origin of nMCC-UP is still debated. Together with the spontaneous regression of a skin primary tumor +/− distant metastasis [30,31], as previously reported in melanoma [32], a further theory addresses the possible nodal primary site [33] as similar to that of melanoma [34]. Despite this, the main pathological differential diagnosis of nMCC-UP is with small cell lung cancer (SCLC), which is TTF-1+, CK-7+, and CK-20 negative [35]. In our series, all cases were TTF-1 negative (100%), whereas CK20 was positive in around 90% of cases. Furthermore, nMCC-UP specimens do not express S-100 and CD45/CD20, which are present in melanoma and lymphoma, respectively [13], while CD99 could help to distinguish MCC from a primitive neuroectodermal tumor (PNET) [36]. Notably, in 16 out 55 patients in our dataset, the original histological diagnosis was different from MCC, mainly NEC, and it changed to MCC throughout the dedicated pathology review. This supports the belief that nMCC-UP is an entity more commonly diagnosed in NEN-referral centers, rather than in skin cancer-referral centers.

Cancers of an unknown primary site (CUP) represent less than 5% of all cancers [37]. Among them, cases of nodal metastasis are only present in a single regional basin with no evidence of a primary site, and no distant metastases have been previously described as related to several types of malignancies [38,39,40]. In this context, nodal MCC-UP represents around 4% of all MCC patients, but up to 40% of those with cN1 [27].

In our study, the 5-year CSS rate was 84.4% for the period of 2010–2019 compared to 50.0% for the period of 1996–2009 (Figure 1c). Several reasons may be adduced, including the establishment of a system in multidisciplinary management, a more frequent therapeutic decision such as the stage IIIB MCC-KP site or, finally, a different procedure for patient selection.

Virus-positive MCC has been reported to have a better prognosis compared with MCC-VN, both KP and UP [41,42] and nMCC-UP [28]. This could be one of the reasons behind the relatively good prognosis of our series as around 90% were MCC-VP. Merkel cell carcinoma UP was reported as MCC-VP more commonly than MCC-KP; in addition, it has been shown that MCC-VP can originate from the derma, whereas MCC-VN can originate from the epidermis [43].

Radical surgery of both primary and nodal tumors has been reported to improve survival compared with RT in stage III MCC with known primary site [44]. However, it is not clear if this approach is beneficial for nMCC-UP as well. Our results suggest that “adjuvant” RT, after LND, can favorably impact RFS and, partially, CSS. The evidence supporting this data is controversial, and some studies report inconsistency regarding RT benefits for OS in stage III MCC [45].

The National Comprehensive Cancer Network (NCCN) 2022 guidelines (Merkel cell carcinoma, Version 2.2022—21 April 2022) recommends the same management of the nodal basin for stage III MCC-KP and nMCC-UP. This is in line with what was suggested for occult breast cancer (Breast cancer, Version 4.2022—21 June 2022).

In our series, 65.5% of patients received curative surgical treatment with LND, 25.4% received palliative therapy, and 9.0% received no therapy beyond an excisional biopsy. Among these latter five patients, two had a surprisingly long DFS. One of these patients with MCC-VN died after 13 years due to other causes, and the other is currently alive with a DFS of 9 years.

Immune checkpoint inhibitors (ICIs) dramatically improved the prognosis of a proportion of patients with advanced MCC. Of our seven patients who experienced systemic relapse, three received an ICI as first-line treatment with good results.

We are aware that our study presents several limitations. The first limitation is the arbitrary clinical management based on personal experience and expertise within our MDT. Second, insufficient information about the characteristics of patients/tumors who underwent curative (LND +/− RT) treatment compared with those who did not receive this approach. Third, the retrospective study design and the long period of observation with relevant changes in clinical management proved limiting. Moreover, due the exploratory nature of the study, we considered many factors as potential predictors of cancer recurrence or cancer survival, and our analysis is subject to multiple testing. We did not present adjusted *p*-values for multiple testing in the tables; however, the threshold of statistical significance using the Bonferroni adjustment method was equal to *p* < 0.003. On the other hand, a potential strength is the large number of cases for a single-center series in the context of a very rare clinical entity, which contributes to increased awareness and knowledge about this unique subset of MCC patients.

## 5. Conclusions

Nodal MCC-UP represents a debated entity and its clinical management is challenging. Our study suggests that a curative approach may be beneficial, as recommended in the main guidelines, with a potential role for “adjuvant radiotherapy”. However, our study was not conceived of as conclusive; therefore, the results should be carefully interpreted and should be considered descriptive in nature. Due to the extreme rarity of this entity, an MCC-dedicated MDT, evaluating all the aspects from histologic diagnosis to treatment selection should be a key point in MCC care. Future investigations on nMCC-UP should include a prospective centralization of pathology, imaging, and clinical data, together with a detailed study of tissue and blood biomarkers in order to stratify different subgroups of patients in terms of prognosis.

## Figures and Tables

**Table 1:** Characteristics and diagnostic workup of patients with nMCC-UP.

	All	1996–2009	2010–2019	*p* Value
	N (%)	N (%)	N (%)	
Total	55 (100.0)	22 (100.0)	33 (100.0)	
Age				
Median (range)				
<50	6 (10.9)	3 (13.6)	3 (9.1)	
50–59	14 (25.5)	7 (31.8)	7 (21.2)	
60–69	21 (38.2)	7 (31.8)	14 (42.4)	
70+	14 (25.5)	5 (22.7)	9 (27.3)	0.76
Sex				
Female	19 (34.5)	5 (22.7)	14 (42.4)	
Male	36 (65.5)	17 (77.3)	19 (57.6)	0.16
Nodal basin				
Inguinal	43 (78.2)	16 (72.7)	27 (81.8)	
Axillary	9 (16.4)	5 (22.7)	4 (12.1)	
Neck	3 (5.5)	1 (4.5)	2 (6.1)	0.68
Previous cancer				
No	45 (81.8)	16 (72.7)	29 (87.9)	
Yes	10 (18.2)	6 (27.3)	4 (12.1)	0.17
Time between LN appearance and diagnosis				
<1 months	11 (26.2)	4 (30.8)	7 (24.1)	
1–3 months	16 (38.1)	3 (23.1)	13 (44.8)	
≥3 months	15 (35.7)	6 (46.2)	9 (31.0)	0.45
^18^FDG-PET/CT				
Not done	15 (27.3)	9 (40.9)	6 (18.2)	
Negative	12 (21.8)	1 (4.6)	11 (48.5)	
Positive	28 (50.9)	12 (54.6)	16 (33.3)	0.02

Time between LN appearance and diagnosis is missing for 13 patients; FDG-PET/CT is missing for 2 patients; SRI for is missing for 1 patient. LN: lymph node; FDG: fluorodeoxyglucose; PET: positron emission tomography; CT: Computed tomography.

**Table 2:** Treatment.

	All	1996–2009	2010–2019	*p* Value
	N (%)	N (%)	N (%)	
Lymph node dissection				
No	19 (34.5)	14 (63.6)	5 (15.2)	
Yes	36 (65.5)	8 (36.4)	28 (84.8)	0.0004
LND−	11 (20.0)	2 (9.1)	9 (27.3)	
LND+	25 (45.5)	6 (27.3)	19 (57.6)	0.0009
Lymph nodes removed				
median (range)	18 (5–50)	13 (5–28)	20 (8–50)	0.11
LN+/LN tot ratio				
mean ± SD	0.21 ± 0.28	0.18 ± 0.34	0.21 ± 0.26	0.74
Radiotherapy				
No	19 (34.5)	10 (45.5)	9 (27.3)	
Adjuvant	24 (43.6)	5 (22.7)	19 (57.6)	
Palliative	10 (18.2)	7 (31.8)	3 (9.1)	0.01
Chemotherapy				
No	30 (54.5)	4 (18.2)	26 (78.8)	
Adjuvant	10 (18.2)	8 (36.4)	2 (6.1)	
Palliative	14 (25.5)	10 (45.5)	4 (12.1)	<0.0001
CT Regimen				
CDDP + VP-16	8 (14.5)	2 (9.1)	6 (18.2)	
DTIC-adriamycin + 5FU	1 (1.8)	1 (4.5)	-	
CBDCA + VP16	14 (25.5)	14 (63.6)	-	
VP-16	1 (1.8)	1 (4.5)	-	0.0002
Overall treatment				
LND + RT (±ChT)	24 (43.6)	5 (22.7)	19 (57.6)	
LND (±ChT)	10 (18.2)	3 (13.6)	7 (21.2)	
No LND *	19 (34.5)	14 (63.6)	5 (15.2)	0.001

Radiotherapy is missing for 2 patients; chemotherapy is missing for 1 patient. * 10 patients received combined RT + CT, 3 patients received CT alone and 6 patients received no treatment. LND: lymphnode dissection; DTIC: dacarbazine; FU: fluorouracil; CDDP: cisplatin; CBDCA: carboplatin; VP-16: etoposide; ChT: chemotherapy; RT: radiotherapy.

**Table 3:** Univariate analysis.

Univariate Analysis	Relapse Free Survival		Cancer Specific Survival	
	HR (95% CI)	*p*-Value	HR (95% CI)	*p*-Value
Year of diagnosis				
2010–2020	1.00		1.00	
1996–2009	2.88 (1.25–6.62)	0.01	3.99 (1.27–12.5)	0.02
Age				
<50	1.00		1.00	
50–59	0.31 (0.07–1.41)	0.13	0.46 (0.06–3.26)	0.44
60–69	1.06 (0.33–3.35)	0.92	1.38 (0.29–6.71)	0.69
70+	0.72 (0.20–2.56)	0.61	1.10 (0.20–6.03)	0.91
Sex				
Male	1.00		1.00	
Female	0.91 (0.38–2.19)	0.83	0.47 (0.13–1.66)	0.24
Site				
Inguinal	1.00		1.00	
Axillar	1.50 (0.56–4.07)	0.42	1.27 (0.35–4.56)	0.71
Neck	0.81 (0.11–6.10)	0.84	1.66 (0.21–13.0)	0.63
Previous cancer				
No	1.00		1.00	
Yes	0.76 (0.26–2.23)	0.62	0.91 (0.26–3.25)	0.89
Time between LN appearance and diagnosis				
<1 month	1.00		1.00	
1–3 months	0.51 (0.14–1.92)	0.32	0.28 (0.05–1.51)	0.14
≥3 months	1.40 (0.44–4.43)	0.57	0.80 (0.20–3.21)	0.75
CK-20				
Negative	1.00		1.00	
Positive	0.98 (0.29–3.31)	0.98	2.04 (0.27–15.6)	0.49
Ki-67				
<80%	1.00		1.00	
80%	0.47 (0.16–1.40)	0.17	0.29 (0.06–1.45)	0.13
≥80%	1.09 (0.42–2.83)	0.86	1.42 (0.46–4.41)	0.54
LTA				
Negative	1.00		1.00	
Positive	No event	0.04 *	No event	0.12 *
Neurofilament				
Negative	1.00		1.00	
Positive	3.44 (0.46–25.6)	0.23	2.20 (0.29–16.8)	0.47
Lymph node dissection (LND)				
No	1.00		1.00	
Yes	0.23 (0.10–0.52)	0.0004	0.17 (0.05–0.53)	0.002
LND−	No event	0.0002 *	No event	0.004 *
LND+	0.36 (0.16–0.82)	0.02	0.26 (0.08–0.83)	0.02
Radiotherapy				
None	1.00		1.00	
Adjuvant	0.12 (0.03–0.42)	0.001	0.19 (0.04–0.91)	0.04
Palliative	1.06 (0.44–2.57)	0.90	1.48 (0.50–4.42)	0.48
Chemotherapy				
None	1.00		1.00	
Adjuvant	0.92 (0.25–3.45)	0.91	1.89 (0.32–11.3)	0.48
Palliative	4.25 (1.78–10.2)	0.001	8.84 (2.42–32.2)	0.001
Cisplatin + etoposide	2.16 (0.66–7.04)	0.20	5.01 (1.01–24.8)	0.049
DTIC-adria + 5FU	15.0 (1.65–137.)	0.02	11.2 (1.14–110.)	0.04
CBDCA + VP16	2.36 (0.93–5.97)	0.07	4.94 (1.28–19.1)	0.02
Etoposide	30.8 (2.94–322.)	0.004	35.6 (3.18–399.)	0.004
Overall treatment				
LND + RT (±CT)	1.00		1.00	
LND (±CT)	6.60 (1.65–26.5)	0.008	2.69 (0.38–19.1)	0.32
No LND *	9.65 (2.78–33.4)	0.0004	8.23 (1.82–37.2)	0.006

* Log-rank test.

**Table 4:** Multivariable analysis.

	Relapse Free Survival		Cancer Specific Survival	
	HR (95% CI)	*p*-Value	HR (95% CI)	*p*-Value
Year of diagnosis				
2010–2019	1.00		1.00	
1996–2009	1.32 (0.50–3.47)	0.57	1.32 (0.38–4.58)	0.66
Overall treatment				
LND + RT (±CT)	1.00		1.00	
LND (±CT)	6.34 (1.57–25.5)	0.009	2.36 (0.33–16.9)	0.39
No LND *	7.80 (2.13–28.6)	0.002	5.15 (1.08–24.6)	0.04
Adjuvant/palliative CT				
No	1.00		1.00	
Yes	1.31 (0.51–3.41)	0.58	2.87 (0.72–11.5)	0.14

* Log-rank test.

## Data Availability

The data presented in this study are available on request from the corresponding authors.

## Supplementary Materials

The following supporting information can be downloaded at: https://www.mdpi.com/article/10.3390/cancers14194777/s1, Table S1. Tumor tissue biomarkers. Table S2. Main published case series of nMCCUP.
