# Supplementary material for: Nodal Merkel Cell Carcinoma with Unknown Primary Site and No Distant Metastasis: A Single-Center Series
Source: Cancers (Basel). 2022 Sep 29;14(19):4777. doi: 10.3390/cancers14194777 (PMC9563944; doi:10.3390/cancers14194777)
Supplement: Supplementary file 1 [file cancers-14-04777-s001.zip › cancers-1889940-supplementary.pdf]

cancers MDPI

Supplementary Materials:

Table S1. Tumor tissue biomarkers.

	All N (%)	1996–2009 N (%)	2010–2019 N (%)	*p* Value
**TTF 1**				
Positive	-	-	-	
Negative	44 (80.0)	21 (95.5)	23 (69.7)	-
**CK-20**				
Positive	49 (89.1)	17 (77.3)	32 (97.0)	
Negative	6 (10.9)	5 (22.7)	1 (3.0)	**0.03**
**Neurofilament**				
Positive	35 (63.6)	20 (90.9)	15 (45.5)	
Negative	6 (10.9)	2 (9.1)	4 (12.1)	0.39
**Ki-67**				
<80%	17 (30.9)	8 (36.4)	9 (27.3)	
80%	16 (29.1)	7 (31.8)	9 (27.3)	
>80%	16 (29.1)	5 (22.7)	11 (33.3)	0.67
**LTA**				
Positive	44 (80.0)	17 (77.3)	27 (81.8)	
Negative	6 (10.9)	2 (9.1)	4 (12.1)	1.00
**TTF 1**				
Positive	-	-	-	
Negative	45 (80.0)	21 (95.5)	24 (69.7)	-
**CK-20**				
Positive	49 (89.1)	17 (77.3)	32 (97.0)	
Negative	6 (10.9)	5 (22.7)	1 (3.0)	**0.03**
**NF**				
Positive	36 (63.6)	20 (90.9)	16 (45.5)	
Negative	6 (10.9)	2 (9.1)	4 (12.1)	0.39
**Ki-67**				
<80%	18 (30.9)	8 (36.4)	10 (27.3)	
80%	16 (29.1)	7 (31.8)	9 (27.3)	
≥80%	16 (29.1)	5 (22.7)	11 (33.3)	0.67
**LTA**				
Positive	45 (80.0)	17 (77.3)	28 (81.8)	
`	6 (10.9)	2 (9.1)	4 (12.1)	1.00

TTF1 is missing for 11 patients, NF for 14, Ki-67 for 6, LTA for 5. TTF-1: thyroid transcription factor; CK: cytokeratine; NF: neurofilament; LTA: large T antigen.

Table S2. Main published case series of nMCCUP.

Ref.	Author	Center	Period	N. pts with Occult Primary	Total N. pts	Median/Mean Age (y)	Males %	Most Common Site	LND n(%)	mOS y	mFup y
[14]	Foote, 2011	Australia, 3 tertiary centers	1985–2010	36	91	74	69	H&N	23 (64)	5.3	4.3
[12]	Deneve, 2012	Tampa	1998–2010	38	321	67	79	H&N	29 (76)	8.6	2.1
[15]	Tarantola, 2012	Mayo Clinic, Rochester	1981–2009	23	n.r.	66	87	inguinal	10 (39)	1.6	2.1
[8]	Chen, 2013	Fox Chase, Philadelphia	1996–2011	16	50 st III	67	69	H&N	8 (50)	not reached	NR
[16]	Haymerle, 2014	Vienna General Hospital	2002–2011	8	57	67	60	inguinal	3 (40)	NR	2.8
[17]	Pan, 2014	Duarte,CA,USA	2000–2011	22	n.r.	65	80	H&N	10 (45)	NR	2.0
N.A.	Fazio, 2022	IEO, Milan	1996–2019	55	307	63	60	inguinal	36 (65.4)	NR	4.3
